# Effects of mental fatigue on psychophysiological responses, kinematic variables and technical actions in small-sided soccer games: a time course analysis

**DOI:** 10.3389/fpsyg.2025.1654701

**Published:** 2025-10-02

**Authors:** Yusuf Soylu, Ersan Arslan, Neslihan Akçay, Mustafa Sakir Akgul, Bulent Kilit, Thiago Ribeiro Lopes, Dalton de Lima-Junior

**Affiliations:** ^1^Faculty of Sport Sciences, Tokat Gaziosmanpasa University, Tokat, Türkiye; ^2^Hasan Dogan Faculty of Sports Sciences, Karabuk University, Karabuk, Türkiye; ^3^Universidade Federal de São Paulo, São Paulo, Brazil; ^4^Department for Life Quality Studies, University of Bologna, Bologna, Italy

**Keywords:** mental fatigue, game-based training, cognitive fatigue, football, game skill, game manipulation

## Abstract

The study compares the effects of different cognitive task durations to induce MF on 4-a-side small-sided soccer games (SSGs) in psychophysiological responses, kinematic profile, and technical performance. Sixteen young soccer players (age: 15.2 ± 0.4 years) randomly performed the mental fatigue (MF) conditions (30-, 45-, and 60-min Stroop Task) and CON sessions (30-, 45-, and 60-min documentary) immediately before a 4-a-side SSGs (4 × 4 min, and 4 min rest). Kinematic profiles were analysed with GPS. Heart rate (HR), the rating of perceived exertion (RPE), MF visual analogue scale, enjoyment, and technical performance were assessed during SSGs. The manipulation checks revealed that players experienced increased MF levels in all MF inducement durations compared with the CON [30-min, CI_95%diff_ = 5.40 (3.20 to 7.50), *p* < 0.001; 45-min, CI_95%diff_ = 8.30 (6.00 to 10.50), *p* < 0.001; and 60-min, CI_95%diff_ = 12.3 (10.00 to 14.50); *p* < 0.001]. Technical performance varied with the duration of MF exposure. Successful pass presented higher values for the CON condition in the 30-min [CI_95%diff_ = 0.68 (0.21 to 1.16); *p* = 0.035], but remained similar for the 45- [CI_95%diff_ = −0.45 (−1.17 to 0.26); *p* = 0.523], and 60-min [CI_95%diff_ = 0.67 (−0.08 to 1.43); *p* = 0.401]. For the unsuccessful pass the CON condition presented lower values in the 30-min [CI_95%diff_ = −2.36 (−2.89 to −1.83); *p* < 0.001] and 60-min [CI_95%diff_ = −2.80 (−3.63 to −1.97); *p* < 0.001], but remained similar for the 45- [CI_95%diff_ = −0.47 (−1.03 to 0.09); *p* = 0.413]. Regarding the one-touch pass, the CON condition presented higher values in the 30- [CI_95%diff_ = 0.63 (0.31 to 0.94); *p* < 0.001], 45- [CI_95%diff_ = 2.21 (1.76 to 2.64); *p* < 0.001], and 60-min [CI_95%diff_ = 1.73 (1.26 to 2.19); *p* < 0.001]. The findings of this study show that the different durations of cognitive tasks significantly affected several internal load metrics and technical performance, but not external load metrics. Understanding the effects of different cognitive task durations during SSGs allows coaches and sports scientists to customize training and recovery programs better, potentially improving player performance.

## Introduction

Soccer, one of the most popular sports in the world, combines physicality, psychological, and mental skills (Clemente et al., 2022). Therefore, many coaches and soccer scientists have focused on training methods that simulate competition demands (Arslan et al., 2021). SSGs have transformed soccer training over the past two decades (Ortiz et al., 2023). SSGs are widely practiced to improve team performance behaviours, increase player interactions, while maintaining the game’s structure (Clemente et al., 2022; Silva et al., 2015). They are categorized as skill-based, game-based, or conditioning-based training approaches, reflecting the players’ or team’s requirements of physical, technical/tactical capacities (Hammami et al., 2018; Hill-Haas et al., 2011). In contrast to traditional training demands, SSGs provide movement patterns, technical-tactical awareness, and high-intensity activities in a more enjoyable, effective, and time-efficient manner (Los Arcos et al., 2015; Moran et al., 2019). However, psychophysiological factors, including coaching encouragement, task constraints, and different MF levels might affect SSGs performance and consequently its adaptations (Soylu et al., 2023b; Soylu et al., 2022; Younesi et al., 2021).

MF is a psychobiological state that arises during demanding cognitive tasks, resulting in an acute feeling of tiredness and/or a decreased performance capacity (Habay et al., 2021; Van Cutsem et al., 2017). Four categories affected by MF were identified: physical, technical and tactical, physiological, and perceptual–cognitive parameters (Smith et al., 2016). MF has already been studied within the context of soccer. Previous studies (Ferreira et al., 2024; de Fortes et al., 2023; Kunrath et al., 2020a; Ponce-Bordón et al., 2022) demonstrated that MF significantly impacted players’ decision-making abilities and visual search patterns and led to decreased accuracy, slower reaction times, and game-based technical performance, including shooting, passing, and ball CON. Therefore, understanding the impact of MF empowers coaches and practitioners to implement strategies that manage and mitigate its effects, ultimately optimizing performance on the field.

Recent studies have indicated the task-dependent nature of MF, with demanding cognitive tasks inducing high fatigue levels in a fraction of the time needed for less demanding ones (Boat et al., 2020; Brown et al., 2020). However, previous studies have mainly focused on a fixed duration (tasks requiring sustained attention, working memory, and response inhibition for 30 min) for cognitive tasks that trigger MF and ignored differences in players’ responses to different durations of cognitive effort (Pageaux and Lepers, 2018; Van Cutsem et al., 2017). Brown and Bray (2017) indicated a dose–response relationship between cognitive task duration and the severity of MF, leading to potential task incompletion. Moreover, the evidence regarding the dose–response relationship remains unclear; therefore, more research is needed (Dallaway et al., 2022). Given the evidence that MF can detrimentally affect players’ decision-making and physical performance, particularly during SSGs (González-Víllora et al., 2022; Kunrath et al., 2020a), investigating the differential cognitive duration becomes imperative for optimizing player performance and training strategies (Badin et al., 2016; Filipas et al., 2021a; Kunrath et al., 2020b; Soylu et al., 2022; Trecroci et al., 2020).

MF is known to impair sport performance, with its influence varying depending on task duration and cognitive demands (Van Cutsem et al., 2017). Early studies using long manipulations (≥30–90 min) such as Stroop or vigilance tasks consistently impaired endurance performance and suggested a 30-min threshold (Kunrath et al., 2020b). However, more recent work challenges this assumption, showing that short but demanding tasks can impair performance within 15–20 min (Brown et al., 2020; Dallaway et al., 2022) while some longer tasks produce similar effects (Bian et al., 2025b; Fortes et al., 2019; Marcora et al., 2009). Meta-analyses confirm that effect sizes range from small to moderate depending on task type, duration, and performance domain (Brown et al., 2020; Van Cutsem et al., 2017). In sport contexts, cognitive load has been shown to affect technical performance and decision-making in SSGs (Badin et al., 2016), suggesting that MF’s impact depends on both duration and task difficulty (O’Keeffe et al., 2020). Our study investigated this dose–response pattern by comparing 30, 45, and 60 min of Stroop tasks before SSGs to determine whether longer exposure increases performance decrements in young soccer players. Given the critical need to clarify the specific effects of varying fatigue durations on performance in SSGs, such insights would allow coaches and sports scientists to better tailor training and recovery programs, ultimately enhancing player performance and well-being. Therefore, this study aimed to examine the effects of three different durations of MF inducement (30, 45, and 60 min) on young soccer players’ psychophysiological, kinematic, and technical performance in SSGs.

## Methods

### Experimental approach

**Figure 1:**
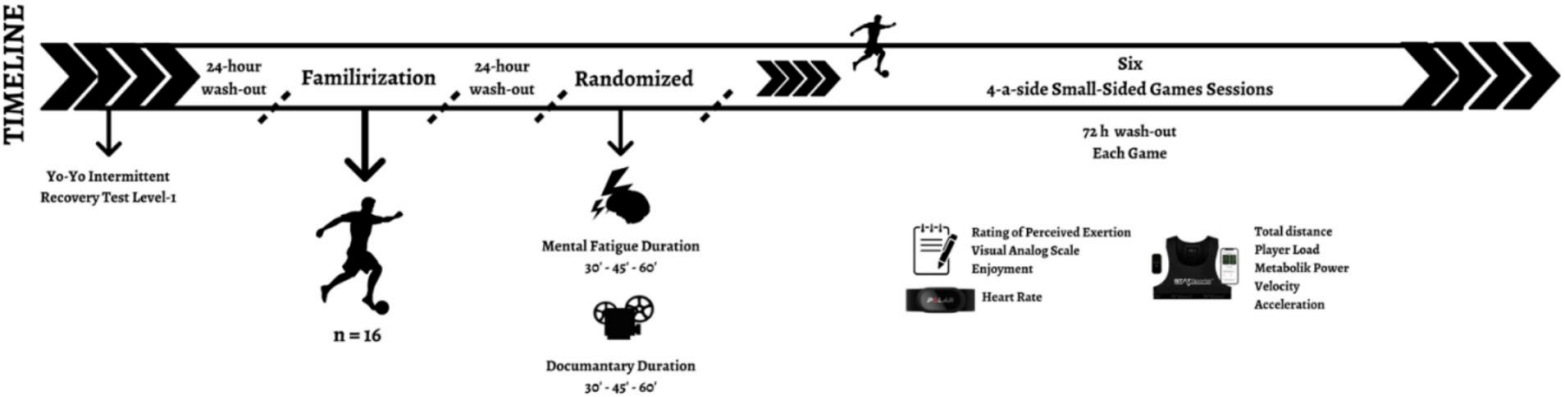
Study design.

A randomized and counterbalanced crossover design with six experimental sessions assessed the effects of different cognitive task durations on the 4-a-side SSGs. The sessions comprised one familiarization, MF interventions (30-, 45-, and 60-min Stroop Task), and CON sessions (30-, 45-, and 60-min documentary) as seen in Figure 1. Players within a given SSGs bout were exposed to the same pre-SSGs condition (i.e., all fatigued or CON), ensuring that mentally fatigued players never competed against non-fatigued players. The design was a within-subjects crossover, with each participant experiencing all six conditions in balanced and counterbalanced order. Trials were conducted at least 72 h apart to allow recovery and minimize the potential negative effects of fatigue (Nedelec et al., 2012). Additionally, all SSGs performed mini-goals to simulate the physiological demands with offensive and defensive tactics used on the field. MF intervention and SSGs sessions were conducted on a natural grass pitch at a similar time to ensure the chronobiological characteristics (Drust et al., 2005). Before all sessions, players were instructed to avoid moderate- to high-intensity exercise, drink alcohol and caffeine for 24 h, and sleep 7–8 h.

### Participants

We performed *a priori* power analysis using G*Power 3.1. Based on previous studies that have examined how MF affects the technical and physiological performance of soccer players (Badin et al., 2016). We determined that a sample size of n = 14 was necessary. Our study included 16 players, exceeding the required number, thereby ensuring adequate statistical power. Sixteen amateur young male soccer players (age: 15.2 ± 0.4 years, body height: 172 ± 4 cm, body mass: 58.43 ± 5.35 kg; body mass index: 19.69 ± 1.59; body fat % 11.54 ± 3.77) participated in this study during the 2023–2024 pre-season period. Participants were categorized as Tier 2 (trained/developmental) based on their weekly training and competition schedules following McKay et al. (2021) Classification Framework. As the inclusion criteria, each player should have a training workload of > 4 training units per week and have been involved in soccer training and league matches for more than 4 years. In case of injuries, players were excluded from the investigation. Before the beginning of the study, players were fully informed about the procedures and signed an informed consent form. The Tokat Gaziosmanpasa University Research Ethics Committee (26.12.2023/20-06) approved the study conducted in accordance with the Declaration of Helsinki.

### Procedures

During the familiarization session, anthropometric values were measured using the bioelectrical impedance measurement (BC-418, Tanita, Tokyo, Japan). The Yo-Yo Intermittent Recovery Test Level-1, a reliable and acoustically CONled progressive test to assess players’ aerobic fitness, which comprises repeated 20 m runs back and forth between the starting, turning, and finishing lines (Bangsbo et al., 2008), was performed. Based on the distances covered in the test, the SSGs teams were ranked to avoid an imbalance in the aerobic fitness levels of the SSGs teams. Before the SSGs, players performed a 15-min warm-up, including jogging, dynamic stretching targeted at the upper and lower limbs, mobility and agility exercises, and soccer-specific ball actions. The game field was a 25 m x 32 m pitch, a familiar size commonly used in training sessions for all participating players. The SSGs were conducted under verbal encouragement and motivation from UEFA-licensed coaches, and balls were placed on the field side for a high play rhythm.

### MF protocols

The MF intervention involved watching a documentary (30-, 45-, and 60-min) to induce cognitive fatigue, as demonstrated in previous studies (Faro et al., 2022; Filipas et al., 2021b). The athletes arrived at the club facilities and watched the documentary film prepared in the meeting room for training. Participants completed the cognitively demanding task in a CONled environment (quiet room) with consistent supervision from the same researchers. Neutral documentaries (Marcora et al., 2009; Moreira et al., 2018) are established CON stimuli for MF studies due to their minimal effect on cognition (L. S. Fortes et al., 2019; Lopes et al., 2020) and brain correlates (Franco-Alvarenga et al., 2019). To assess MF, a 100-mm visual analogue scale (VAS) was also reported by players after each SSGs bout. These highly validated and reliable scales were frequently used to measure the level of physical and MF according to previous studies (Faro et al., 2022; Soylu et al., 2022).

### Randomization

#### Sequence generation, allocation concealment, and blinding

The participants were randomly and counterbalancedly assigned to six experimental conditions. They were randomized using a random number table. The allocation was concealed from the researcher who enrolled and assessed participants, and only after the baseline procedures the allocation took place. Blinded researchers performed baseline measurements, and researchers who supervised the SSGs were blinded to the conditions in which the participants were inserted.

### SSGs sessions

Anthropometric values were measured using bioelectrical impedance measurements (BC-418; Tanita, Tokyo, Japan). The Yo-Yo Intermittent Recovery Test Level-1 (Yo-Yo IR1), a reliable progressive test for aerobic fitness assessment (Bangsbo et al., 2008), was conducted. Since external load and tempo in SSGs are closely related to aerobic conditioning, we balanced the teams according to Yo-Yo IR1 to minimize running-based imbalances. Therefore, deviations caused by the running capacity were reduced in the technical performance comparisons. In addition, to maintain technical-tactical balance, players were assigned similar roles/positions by the same coach. The players performed a standardized 15-min warm-up including jogging, dynamic stretching, mobility and agility drills, and soccer-specific ball actions before each SSGs. The SSGs (4-a-side with mini-goals) were performed on a natural grass outdoor pitch of 25 × 32 m (length × width), providing a relative playing area of ~ 100 m per player. This pitch dimension matches the formats commonly used in youth soccer training (Soylu et al., 2022). Each session comprised four 4-min bouts with 4-min passive recovery periods, totaling 16 min of play. Standard soccer rules were applied, with the exception of the no-offside rule. The coaches provided verbal encouragement without technical or tactical feedback (Skala and Zemková, 2023). Several balls were placed around the pitch for quick restart. The players were familiar with the SSGs format from their regular training programs.

### External load

During experimental and CON sessions in SSGs, global positioning system (GPS) devices with a 10 Hz sampling rate (Catapult Vector S7, Catapult Sports, Australia) were utilized to monitor external loads and have been validated for reliability and accuracy (Crang et al., 2022), allowing for accurate monitoring of an athlete’s movements and calculation of distance covered based on changes in position over time. The metrics derived from each of the Catapult Vector S7 were total distance (m), moderate speed running (14.0–17.9 km·h^−1^), high speed running (18.0–20.9 km·h^−1^), and sprinting (above 21.0 km·h^−1^), total accelerations (>3 m·s^−2^), Playerload and metabolic power. PlayerloadTM, in arbitrary units (au), quantifies the total effort as the square root of the sum of the squared instantaneous changes in three vectors divided by 100. Metabolic Power (W/kg) estimates the equivalent energy cost of running on a flat surface as if on an inclined surface, considering the mechanical work required for acceleration, deceleration, or directional changes (Walker et al., 2016). The players wore the same GPS devices throughout all SSGs sessions, which were placed right between the players’ upper scapulae.

### Internal load

The internal load parameters, including heart rate (HR), average HR (HR_mean_), the rating of perceived exertion (RPE), exercise enjoyment, and visual analogue scale for MF, were recorded during SSGs. During each SSGs intervention, players wore Bluetooth sensors (Polar H10, Kempele, Finland), recording HR data every 5 s and integrating with the GPS data. The RPE provides a valuable evaluation of the players’ subjective experience of the game’s intensity (Foster et al., 2021). RPE was familiarized to players before study commencement so they could assess their in-task RPE during each SSGs. The scale consists of a range from 6 to 20 to indicate a number on the scale that best represents their subjective rating or evaluation. Before initiating the study, every participant received comprehensive training on properly utilizing the RPE. Players’ subjective MF was evaluated using a 100-mm paper visual analogue scale with anchors. The players are typically presented with a horizontal scale or line, with the left terminus designated as zero (0) to indicate “no fatigue” or “absence of tiredness” and the right terminus labeled as one hundred (100) to represent “extreme fatigue” or “maximum tiredness.” The players were instructed to indicate a point on the line corresponding to their perceived MF level (Soylu et al., 2022). Enjoyment levels were determined by how much players enjoyed exercise during and immediately after SSGs that used the exercise enjoyment scale. Raedeke (2007) scale has eight items and is scored on a 7-point scale and is bipolar. Scale was included items such as ‘I enjoyed it—I hated it’ and ‘I disliked it—I liked it’, providing a validated multidimensional measure of enjoyment in exercise contexts. The highest score that can be obtained from the scale is 56, and the lowest score is 8. Soylu et al. (2023a) adapted the scale for the Turkish athletes. RPE, VAS, and enjoyment scores were systematically recorded immediately following each SSGs, within a 30 to 60-s interval after the final whistle.

### Technical performance

Technical activities were recorded in 1080p quality using a Canon HF R806 video camera (Canon, Tokyo, Japan) in each SSGs. E-Analyze Digital Soccer Match Analysis Software was used (Espor Digital, Ankara, Türkiye). Technical performance were coded as follows: successful and unsuccessful pass, interception, and lost ball. The technical performance were assessed by a match and performance analysis coach with more than 3 years of experience.

### Statistical analysis

The Generalized Mixed Models (GLzMM) analyzed the main effects and interaction between condition (i.e., CON x MF) and duration (i.e., 30-, 45-, 60-min) for all the analyzed variables. The GLzMM set up as follows: (a) subjects, condition, duration, and interaction were tested in the model as random effects; (b) duration as the within-subject variable; (c) Gamma or Gaussian distributions with identity link function for model type; (d) condition and duration as factors; (e) Akaike Information Criterion for the better-fit model; (f) Wald chi-square statistics as the model effects; (g) Holm post-hoc for pairwise comparisons; and (h) graphic analysis of the residuals (de Melo et al., 2022; Zeger and Liang, 1986). The analyses were made using JAMOVI v2.5.3.0.

## Results

The participants completed all six conditions, and no adverse events were reported. The present study includes the effects of MF interventions with different durations on psychophysiological, kinematic, and technical performance in 4-a-side SSGs.

### Manipulation check

#### Visual analogue scale

**Figure 2:**
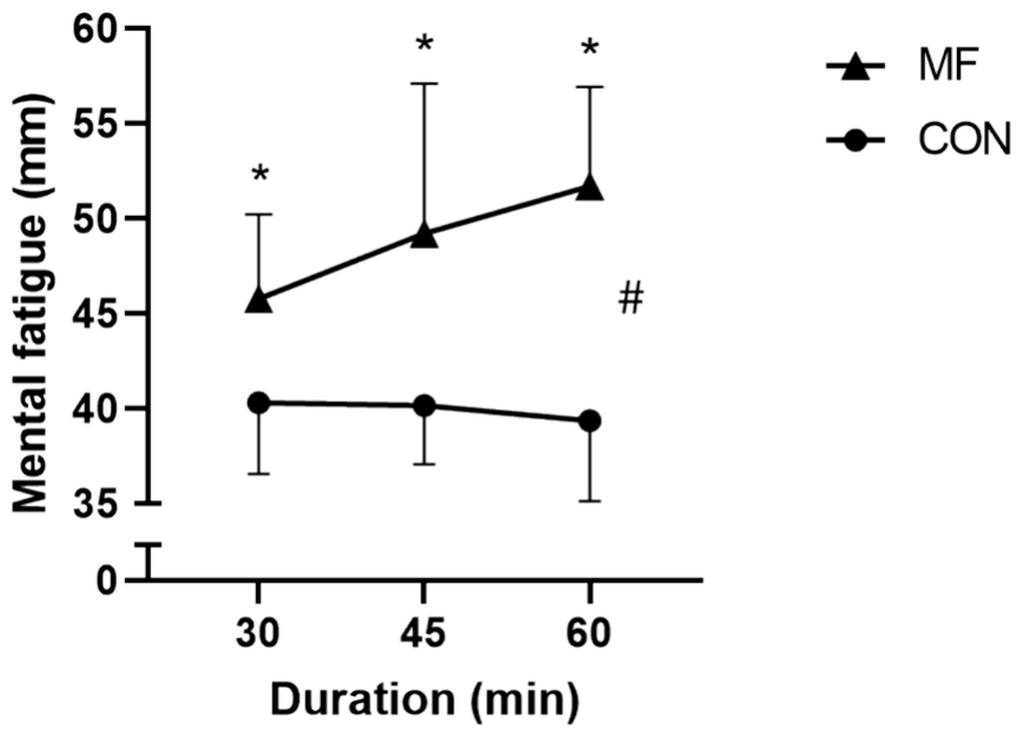
Subjective mental fatigue. MF = mental fatigue; CON = control. # = main effect of condition; * different from CON.

We found a condition [X^2^_(1,45)_ = 169.1; *p* < 0.001] and interaction [X^2^_(1,45)_ = 18.1; *p* < 0.001] effects. The main effect of duration [X^2^_(2,45)_ = 1.7; *p* = 0.42] was not significant (Figure 2). The CON condition was different from MF in the 30- [CON 30 = 40.0 ± 4.0 mm; MF 30 = 46.0 ± 4.0 mm.; CI_95%diff_ = 5.40 (3.20 to 7.50); *p* < 0.001], 45- [CON 45 = 40.0 ± 3.0 mm; MF 45 = 49.0 ± 8.0 a.u.; CI_95%diff_ = 8.30 (6.00 to 10.50); *p* < 0.001], and 60-min [CON 60 = 39.0 ± 4.0 a.u.; MF 60 = 52.0 ± 5.0 a.u.; CI_95%diff_ = 12.3 (10.00 to 14.50); *p* < 0.001].

### Internal load

#### Rating of perceived exertion

**Figure 3:**
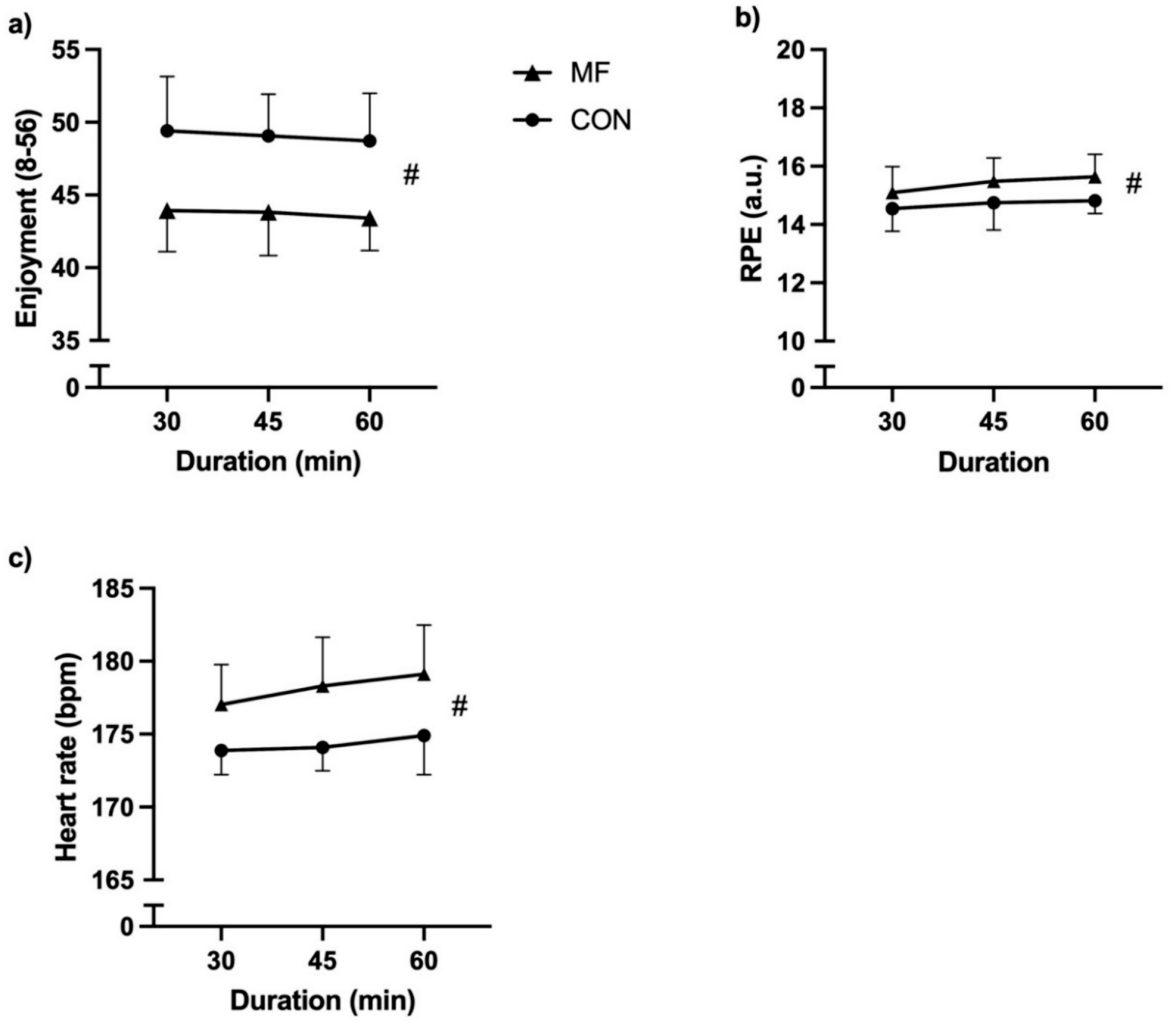
Internal load. (**a**) Enjoyment; (**b**) RPE; (**c**) Heart rate. MF = mental fatigue; CON = control; RPE = rating of perceived exertion. # = main effect of condition.

A condition main effect was found [X^2^_(1,45)_ = 46.5; *p* < 0.001]. However, the main effect of duration [X^2^_(2,45)_ = 1.9; *p* = 0.39] and the interaction effect [X^2^_(2,45)_ = 1.3; *p* = 0.52] were not significant (Figure 3a). The CON condition presented lower values compared to MF [CON = 4.0 ± 0.3 a.u.; MF = 4.9 ± 0.7 a.u.; CI_95%diff_ = −0.69 (−0.90 to −0.50)].

#### Enjoyment

We found a condition main effect [X^2^_(1,45)_ = 221.9; *p* < 0.001]. The main effect of duration [X^2^_(2,45)_ = 0.2; *p* = 0.89] and the interaction effect [X^2^_(2,45)_ = 0.1; *p* = 0.97] were not significant (Figure 3b). For this variable, the CON condition presented higher values compared to MF [CON = 49.1 ± 3.2 a.u.; MF = 43.7 ± 2.6 a.u.; CI_95%diff_ = 5.30 (4.60 to 6.00)].

#### Heart rate

We found a condition main effect [X^2^_(1,45)_ = 150.2; *p* < 0.001]. The main effect of duration [X^2^_(2,45)_ = 2.0; *p* = 0.36] and the interaction effect [X^2^_(2,45)_ = 2.5; *p* = 0.29] were not significant (Figure 3c). For heart rate, the CON condition presented lower values compared to MF [CON = 174.1 ± 2.0 bpm; MF = 178.7 ± 3.2 bpm; CI_95%diff_ = −3.83 (−4.45 to −3.22)].

### External load

#### Player load

**Figure 4:**
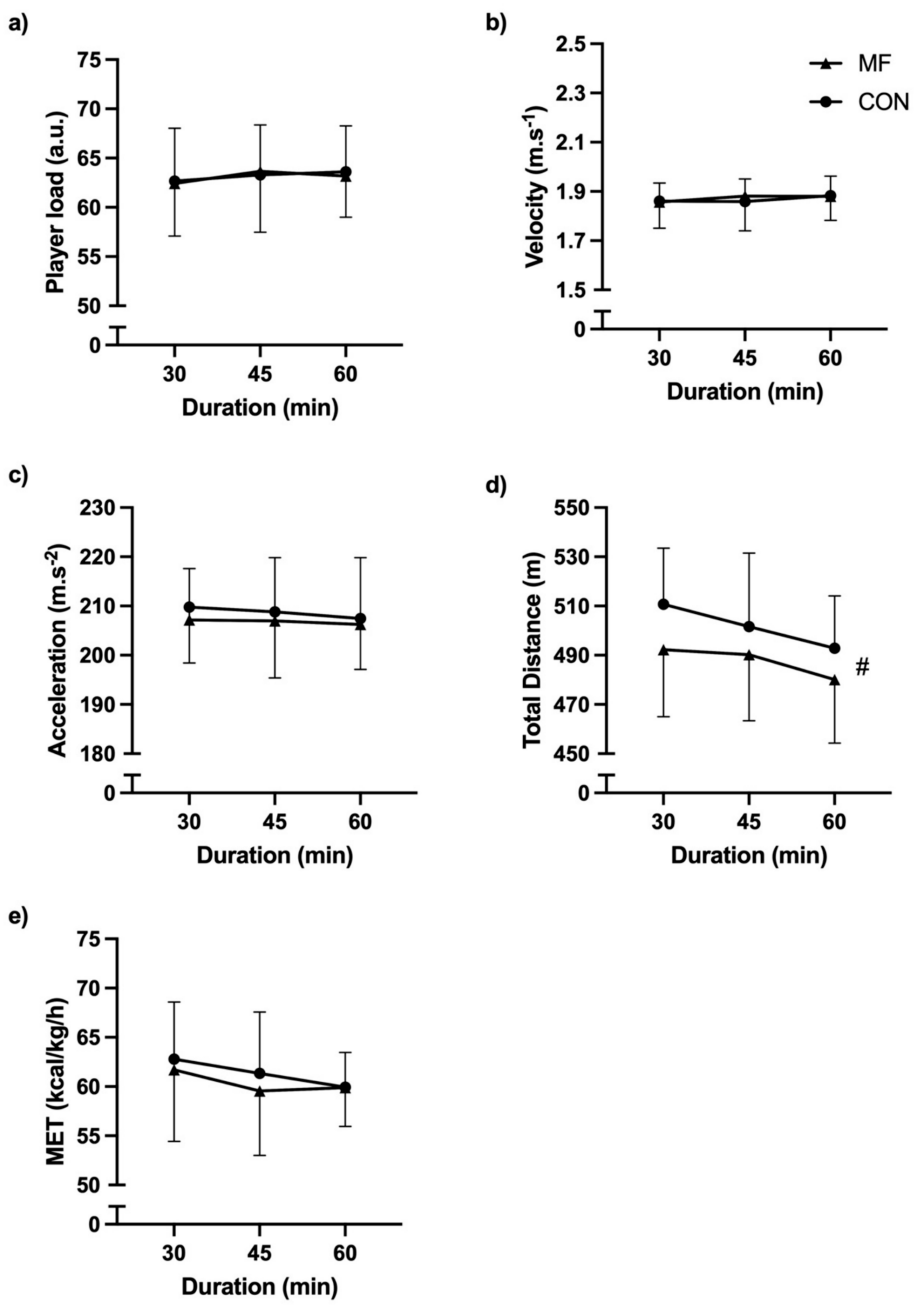
External load. (**a**) Player load; (**b**) Velocity; (**c**) Acceleration; (**d**) Total distance; (**e**) MET. MF = mental fatigue; CON = control; MET = metabolic power. # = main effect of condition.

No effects of condition [X^2^_(1,45)_ = 0.1; *p* = 0.87], duration [X^2^_(2,45)_ = 0.2; *p* = 0.91], or interaction [X^2^_(2,45)_ = 0.8; *p* = 0.67] were found (Figure 4a).

#### Velocity

No effects of condition [X^2^_(1,45)_ = 0.5; *p* = 0.49], duration [X^2^_(2,45)_ = 0.3; *p* = 0.84], or interaction [X^2^_(2,45)_ = 1.3; *p* = 0.52] were found (Figure 4b).

#### Acceleration

No effects of condition [X^2^_(1,45)_ = 2.4; *p* = 0.12], duration [X^2^_(2,45)_ = 0.1; *p* = 0.95], or interaction [X^2^_(2,45)_ = 0.1; *p* = 0.94] were found (Figure 4c).

#### Total distance

We found a condition main effect [X^2^_(1,45)_ = 38.9; *p* < 0.001]. The main effect of duration [X^2^_(2,45)_ = 2.1; *p* = 0.35] and the interaction effect [X^2^_(2,45)_ = 2.0; *p* = 0.36] were not significant (Figure 4d). The CON condition presented higher values compared to MF [CON = 503.0 ± 25.5 m; MF = 488.0 ± 26.6 m; CI_95%diff_ = 14.34 (9.83 to 18.85)].

#### Metabolic power

No effects of condition [X^2^_(1,45)_ = 3.0; *p* = 0.08], duration [X^2^_(2,45)_ = 1.0; *p* = 0.59], or interaction [X^2^_(2,45)_ = 1.4; *p* = 0.49] were found (Figure 4e).

### Technical performance

#### Successful pass

**Figure 5:**
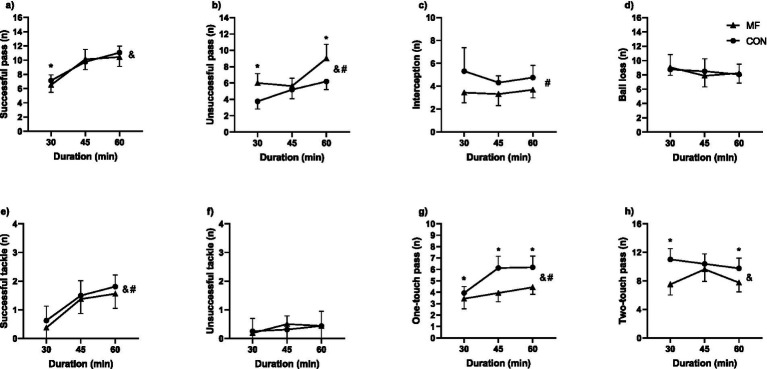
Technical performance. (**a**) Successful pass; (**b**) Unsuccessful pass; (**c**) Interception; (**d**) Ball loss; (**e**) Successful tackle; (**f**) Unsuccessful tackle; (**g**) One-touch pass; (**h**) Two-touch pass. # = main effect of condition; & = main effect of duration; * = different from control.

We found a duration [X^2^_(2,45)_ = 81.3; *p* < 0.001] and interaction [X^2^_(2,45)_ = 2.0; *p* = 0.14] effects, but a condition [X^2^_(1,45)_ = 2.4; *p* = 0.12] effect was not found (Figure 5a). The CON condition presented higher values in the 30-min [CON 30 = 7.1 ± 0.8 n; MF 30 = 6.5 ± 1.0 n; CI_95%diff_ = 0.68 (0.21 to 1.16); *p* = 0.035], but remained similar for the 45- [CON 45 = 9.8 ± 1.7 n; MF 45 = 10.1 ± 1.4 n; CI_95%diff_ = −0.45 (−1.17 to 0.26); *p* = 0.523], and 60-min [CON 60 = 11.1 ± 0.9 n; MF 60 = 10.4 ± 1.3 n; CI_95%diff_ = 0.67 (−0.08 to 1.43); *p* = 0.401].

#### Unsuccessful pass

We found a condition [X^2^_(1,45)_ = 94.7; *p* < 0.001], duration [X^2^_(2,45)_ = 49.1; *p* < 0.001] and interaction effect [X^2^_(2,45)_ = 31.0; *p* < 0.001] (Figure 5b). The CON condition presented lower values in the 30-min [CON 30 = 3.7 ± 0.9 n; MF 30 = 6.0 ± 1.1 n; CI_95%diff_ = −2.36 (−2.89 to −1.83); *p* < 0.001] and 60-min [CON 60 = 6.2 ± 0.9 n; MF 60 = 9.0 ± 1.7 n; CI_95%diff_ = −2.80 (−3.63 to −1.97); *p* < 0.001], but remained similar for the 45- [CON 45 = 5.2 ± 1.1 n; MF 45 = 5.6 ± 0.9 n; CI_95%diff_ = −0.47 (−1.03 to 0.09); *p* = 0.413].

#### Interception

We found a condition [X^2^_(1,45)_ = 33.9; *p* < 0.001] effect, but not a duration [X^2^_(1,45)_ = 3.4; *p* = 0.18] or interaction [X^2^_(2,45)_ = 2.3; *p* = 0.32] effects (Figure 5c). The CON condition presented higher values compared to MF [CON = 4.8 ± 1.4 n; MF = 3.5 ± 0.9 n; CI_95%diff_ = 1.33 (0.88 to 1.77)].

#### Ball loss

No effects of condition [X^2^_(1,45)_ = 0.1; *p* = 0.87], duration [X^2^_(2,45)_ = 1.6; *p* = 0.22], or interaction [X^2^_(2,45)_ = 1.5; *p* = 0.24] were found (Figure 5d).

#### Successful tackle

We found a condition [X^2^_(1,45)_ = 8.0; *p* = 0.007] and duration effect [X^2^_(2,45)_ = 35.8; *p* < 0.001], but not an interaction effect [X^2^_(2,45)_ = 0.3; *p* = 0.72] (Figure 5e). The CON condition presented higher values compared to MF [CON = 1.3 ± 0.7 n; MF = 1.1 ± 0.7 n; CI_95%diff_ = 0.21 (0.06 to 0.35)] as well as the number of successful tackles augmented from 30- to 45- [30-min = 0.5 ± 0.5 n; 45-min = 1.4 ± 0.5 n; CI_95%diff_ = 0.21 (0.06 to 0.35); *p* < 0.001] and 60-min [30-min = 0.5 ± 0.5 n; 60-min = 1.7 ± 0.4 n; CI_95%diff_ = 0.21 (0.06 to 0.35); *p* < 0.001].

#### Unsuccessful tackle

No effects of condition [X^2^_(1,45)_ = 0.1; *p* = 0.87], duration [X^2^_(2,45)_ = 2.1; *p* = 0.35], or interaction [X^2^_(2,45)_ = 0.7; *p* = 0.70] were found (Figure 5f).

#### One-touch pass

We found significant effects for condition [X^2^_(1,45)_ = 156.5; *p* < 0.001], duration [X^2^_(2,45)_ = 29.1; *p* < 0.001], and interaction [X^2^_(2,45)_ = 36.9; *p* < 0.001] (Figure 5g). The CON condition presented higher values in the 30- [CON 30 = 3.9 ± 0.6 n; MF 30 = 3.4 ± 0.9 n; CI_95%diff_ = 0.63 (0.31 to 0.94); *p* < 0.001], 45- [CON 45 = 6.1 ± 1.0 n; MF 45 = 3.9 ± 0.8 n; CI_95%diff_ = 2.21 (1.76 to 2.64); *p* < 0.001], and 60-min [CON 60 = 6.2 ± 1.0 n; MF 60 = 4.4 ± 0.6 n; CI_95%diff_ = 1.73 (1.26 to 2.19); *p* < 0.001].

#### Two-touch pass

We found significant effects for condition [X^2^_(1,45)_ = 79.8; *p* < 0.001] and interaction [X^2^_(2,45)_ = 21.4; *p* < 0.001], but not for duration [X^2^_(2,45)_ = 4.7; *p* = 0.09] (Figure 5h). The CON condition presented higher values for the 30- [CON 30 = 11.0 ± 1.5 n; MF 30 = 7.5 ± 1.4 n; CI_95%diff_ = 3.67 (2.83 to 4.51); *p* < 0.001] and 60-min [CON 60 = 9.8 ± 1.4 n; MF 60 = 7.8 ± 1.3 n; CI_95%diff_ = 2.10 (1.31 to 2.88); *p* < 0.001], but not for the 45- [CON 45 = 10.4 ± 1.4 n; MF 45 = 9.6 ± 1.7 n; CI_95%diff_ = 0.82 (−0.05 to 1.69); *p* = 0.33].

## Discussion

Our study aimed to investigate the effects of MF on psychophysiological, kinematic, and technical performance in young soccer players exposed to different durations of cognitive interventions (30, 45, and 60 min). The technical performance results showed a statistically significant difference in unsuccessful and one-touch or two-touch passes between the MF and CON conditions. Although no significant changes occurred between 30 and 45 min, a notable decline in technical performance, particularly unsuccessful passes, was evident after 60 min of MF. Considerable variations were observed in the one-touch and two-touch passes at 45–60 min of MF. MF significantly increased the HR compared to that in the CON condition. However, exposure duration (30, 45, and 60 min) alone did not considerably affect HR. Although there was a slight increase in the HR as the duration increased, this change was not statistically significant. Additionally, no significant differences were found in the interaction between the MF and duration. For the manipulation check, visual analogue scale results indicated that MF experienced greater intensity at 60 min compared to 30 and 45 min. Similarly, the perception of effort was high in the MF condition, whereas enjoyment presented reduced levels. The results partially agree with our hypotheses.

Skala and Zemková (2023) reported that fatigue from SSGs in youth soccer players impaired agility, decision-making, and explosive strength, while these effects were not strongly reflected in load variables, indicating that sport-specific fatigue may not be quantified by traditional monitoring. This study revealed that internal and external loads affect MF and found differences between HR and distance. This means that when mentally fatigued, the soccer players ran a shorter distance, although they presented higher HR values. The specific result is uncommon in the literature (Penna et al., 2018; Smith et al., 2016), but it could be explained by the higher stress levels caused by a higher-than-normal perception of effort. In previous studies, physiological parameters such as HR, oxygen volume, and cardiac output remained similar between conditions (Marcora et al., 2009; Smith et al., 2016, 2017; Soylu et al., 2022). However, the tasks were until failure, meaning the dynamics are entirely different from SSGs. Our study and Kunrath et al. (2020a, 2020b) presented contradictory numbers regarding the distance covered. However, the authors explained that this might occur due to a lack of positioning and tactical aspects during a soccer match. In our study, our soccer players covered a shorter distance than the CON group when mentally fatigued, following findings from Smith et al. (2016) and Badin et al. (2016).

The technical performance follow the scientific literature. We found effects for pass and tackle, but not interception and ball loss. Throughout the years, we have observed negative MF effects on passing skills (Badin et al., 2016; Smith et al., 2016, 2017; Soylu et al., 2022). Smith et al. (2016) observed a reduced pass accuracy in the Loughborough passing test following the 30-min Stroop task. Similarly, the same group found impaired pass accuracy following a paper version of the Stroop task (Smith et al., 2017). In line with our findings that MF impaired technical performance but not external load variables, Coutinho et al. (2017) showed that MF in young soccer players reduced tactical synchronization and positioning behaviors while not affecting physical activity, indicating that cognitive fatigue primarily disrupts decision-making rather than locomotor demands. Unlike the previous studies, we analysed different types of passes, such as one- and two-touch passes, showing that more prolonged cognitive effort results in enlarged negative effects. As the impact of the task duration seems to impair only the technical performance of passing, we might speculate that the complexity and accuracy level required to perform a successful pass, different from intercepting and tackling, might require more cognitive resources, making it more susceptible to the negative effects of MF. Additionally, several studies (Badin et al., 2016; Soylu et al., 2022) have found reduced tackle success besides impaired accuracy after inducing MF in soccer players. It corroborates our findings, as we observed only a main effect of condition; the duration of the MF task might not alter the tackle results.

For the manipulation check, we noticed an increase in subjective MF and perception of effort. However, enjoyment levels were reduced in the state of MF. Our study follows the scientific literature, showing that prolonged cognitive tasks that induce MF increase the perception of effort (de Lima-Junior et al., 2024). It might be explained by the psychobiological model (de Morree and Marcora, 2015). Additionally, we observed decreased levels of enjoyment during exercise. Previous studies have investigated the association between MF and enjoyment (Gergelyfi et al., 2015; Sansone et al., 2019). Similarly, our study has demonstrated that an increase in perception of effort levels seems to cause a decrease in enjoyment of a task (Gergelyfi et al., 2015). It could be important mainly in amateur and recreational sports, in which adherence is moderated by enjoyment levels (Gergelyfi et al., 2015; Sansone et al., 2019). The reduction in enjoyment due to MF can be physiologically attributed to increased activation of the prefrontal cortex, which heightens perceived effort and diminishes motivational drive through dopaminergic pathways (de Morree and Marcora, 2015; Marcora et al., 2009). This imbalance between the perceived cost and reward results in decreased task enjoyment. Adolescents may be more susceptible to this effect because of the ongoing maturation of their prefrontal and reward systems (Casey et al., 2008).

Subjective measures such as RPE and enjoyment can be influenced by perceptual bias, this bias reflects the psychobiological impact of MF on motivational-affective responses (Pageaux and Lepers, 2018). It could be important mainly in the context of amateur and recreational aspects of sports, in which adherence is an important factor moderated by enjoyment levels (Gergelyfi et al., 2015; Sansone et al., 2019). Finally, it is important to highlight that more time spent on cognitive tasks might cause greater reduction in performance.

Our findings have practical implications for soccer practitioners. Cognitively demanding activities should be scheduled away from SSGs when technical precision is needed, as even short blocks of decision-heavy tasks, such as Footbonaut, can impair soccer skills despite minimal changes in cognitive measures. When training under cognitive stress, practitioners may use soccer-specific tests such as the Loughborough Soccer Passing Test, which effectively elicits MF. The cognitive dose should match session objectives, as task complexity, contextual interference, and adaptivity significantly influence fatigue. Coaches should use appropriate CON tasks and allow standardized recovery before SSGs to avoid confounding effects. Monitoring both subjective indices (RPE, visual analogue scale) and soccer-specific performance indicators provides a complete picture of player readiness.

Although we presented innovative findings that might help the decision-making of coaches and other soccer professionals, our study presents some limitations. Our participants were young male amateur soccer players, so the generalization to other populations should be made cautiously. We controlled the duration of the intervention but not the level of MF it caused individually, which could have altered the results. We lack motivation measurements, which might be an important covariate regarding MF. Then, future studies should explore different levels of athletes and how they react to different cognitive tasks, as well as how the performance is affected by different levels of MF. Our pre-SSGs manipulation used a Stroop task, providing internal CON, but less representativeness than soccer-specific protocols. Following recent studies (Bian et al., 2025a; Weiler et al., 2025), who used the Loughborough Soccer Passing Test and Footbonaut for ecological validity, future studies should employ sport-specific paradigms with CON tasks. While we tested 30-, 45-, and 60-min exposures under Stroop conditions, Bian et al. (2022) have noticed that duration alone may not reflect dose without considering task difficulty. Future studies should examine different durations with ecological inductions to establish the validity of soccer training.

## Conclusion

The present findings demonstrated that varying conditions significantly influenced the participants, particularly concerning internal load and technical performance. Conversely, external loads remained relatively independent of these conditions. Our study demonstrated that the duration of cognitive tasks does not affect the technical aspects of passing in male amateur soccer players. Although we observed the effects of MF on the physical and technical performance of soccer, the impaired performance was observed only for the pass, one-touch pass, and two-touch pass. These results indicate that diverse conditions may modulate players’ cognitive and physical performances. However, the different MF durations did not substantially affect the external loading levels. In this context, assessing internal responses and technical proficiency is particularly important for optimizing players’ performance.

## Data Availability

The datasets presented in this study can be found in online repositories. The names of the repository/repositories and accession number(s) can be found in the article/supplementary material.
